# Chemotherapy in Nonmetastatic Osteosarcoma: Recent Advances and Implications for Developing Countries

**DOI:** 10.1200/JGO.2016.007336

**Published:** 2017-01-18

**Authors:** Sameer Rastogi, Aditi Aggarwal, Akash Tiwari, Vinod Sharma

**Affiliations:** **Sameer Rastogi**, **Akash Tiwari**, and **Vinod Sharma**, All India Institute of Medical Sciences, New Delhi; and **Aditi Aggarwal**, Fortis Hospital, Noida, India.

## Abstract

**Purpose:**

Osteosarcoma (OS) is a relatively chemosensitive primary bone tumor, with the peak age of onset occurring in late childhood and early adolescence. The treatment paradigm of nonmetastatic OS has typically been multimodality therapy, including neoadjuvant and adjuvant chemotherapy with definitive surgery. Over the years, various permutations and combinations of chemotherapeutic agents have been used. However, the majority of recent trials have still used high-dose methotrexate as the backbone, with cisplatin and doxorubicin (MAP). In the last decade, various strategies targeted to improving outcomes in OS have included the addition of a fourth drug to the three-drug MAP regimen, changing therapy according to histopathologic response and the addition of immunotherapies. Through this review, we sought to underscore a few pertinent issues related to chemotherapy in nonmetastatic OS, with special reference to challenges confronted in Indian settings.

**Methods:**

We reviewed the literature, focusing on studies comparing high-dose methotrexate and non–high-dose methotrexate–containing regimens. In addition, this review focuses on non–methotrexate-containing triple-drug therapy.

**Results:**

Although a high-dose methotrexate regimen has become standard of care in developed countries, there are few data to suggest that it is superior to a non–high-dose methotrexate regimen.

**Conclusion:**

Developing countries with lack of infrastructure and logistics for high-dose methotrexate might resort to non–high-dose methotrexate–containing regimens with a simultaneous focus on early detection, decreasing abandonment, multidisciplinary clinics, improved surgery, and meticulous pathologic evaluations.

## INTRODUCTION

Osteosarcoma (OS) is the most common primary malignant bone tumor, usually occurring in the second decade of life. The advent of chemotherapy in nonmetastatic OS has dramatically improved the 5-year event-free survival from < 20% to 60% to 70%, although not much has changed in the last three decades.^1^ The four most active chemotherapeutic drugs used in OS are methotrexate, cisplatin, doxorubicin, and ifosfamide. After an initial period of intense skepticism, high-dose methotrexate (HDMtx) regimens, eg, MAP (methotrexate, adriamycin, and cisplatin), have become standard of care in North America and Europe.^2^ As of now, the question of using non-HDMtx–based regimens is no more pertinent for Western settings and is not a priority for ongoing research.^3^ There exists a lack of clarity in regard to how and which combinations to use, especially in the setting of a resource-limited country such as India, and there is insufficient evidence of these practices. In this article, we focused on the current standard of care vis-a-vis the chemotherapy of practical choice in a resource-constrained environment to provide the best benefit in the interest of patients.

## WHY IS AN HDMtx-CONTAINING TRIPLE REGIMEN (MAP) THE STANDARD OF CARE?

The use of a triple regimen containing HDMtx, doxorubicin, and cisplatin (MAP; as was used in the control arm of the recently published European and American Osteosarcoma Study Group [EURAMOS]-1 trial^4^) is the standard of care in most developed nations. The HDMtx-based regimen is widely used, but it is surprising to note that there are no head-to-head randomized trials proving its superior efficacy to a non-HDMtx–containing regimen.

The history of HDMtx in OS is interesting and is replete with both positive and negative trials. Initial enthusiasm as a result of good response rates and outcomes was dampened by a controlled trial by the Mayo Clinic comparing HDMtx and amputation versus amputation only; this study showed that there was no apparent improvement with the administration of HDMtx.^5^ However, this study had a small sample size of only 38 patients, and the dose of HDMtx used was < 8 gm/m^2^. Subsequently, a two-arm randomized trial, the Multi-Institutional Osteosarcoma Study, used surgical ablation and HDMtx-based chemotherapy (MAP) in one arm and surgical ablation only in the other (control) arm; an unforeseen benefit in the chemotherapy arm was reported.^6^ It is difficult to pinpoint the chemotherapy drug responsible for the success, because all of these drugs have good activity as single agents. The efficacy of HDMtx was finally accepted, but with variable enthusiasm. However, the search continued thereafter for the optimal regimen.

In the only randomized trial conducted to test three drugs versus a two-drug regimen, patients in the European Osteosarcoma Intergroup trial were randomly assigned to a cisplatin plus doxorubicin backbone with or without HDMtx.^7^ There was a disease-free survival benefit with the cisplatin and doxorubicin–containing regimen, but no difference in the overall survival between these regimens. However, this study has been criticized for the inadequate dose of HDMtx (8 gm/m^2^ compared with the current standard dose of 12 gm/m^2^) and lower dose intensity, with fewer cycles of cisplatin and doxorubicin in the arm with HDMtx. In a subsequent European Osteosarcoma Intergroup study, the inclusion of methotrexate in a multidrug arm (HDMtx, doxorubicin, bleomycin, cyclophosphamide, dactinomycin, vincristine, and cisplatin) did not show any advantage compared with two-drug regimens (cisplatin and doxorubicin). This study had extremely poor compliance in the multidrug arm, a suboptimal methotrexate dose (8 gm/m^2^), and overall poor outcomes in both arms. In addition, in the multidrug arm it included drugs such as actinomycin D and bleomycin, which had limited activity in OS.^8^ After the poor outcomes in the latter trial, two-drug regimens went out of favor and were minimally used in further trials.

Because randomized trials were not able to answer this question, the answer was sought through meta-analyses. van Dalen et al^9^ published a Cochrane meta-analysis suggesting that there is not enough evidence to approve or disapprove use of HDMtx in OS. Another meta-analysis by Anninga et al^10^ showed that regimens containing at least three drugs (including methotrexate plus adriamycin plus cisplatin [plus ifosfamide; MAP(Ifo)]) had significantly better outcomes than two-drug regimens, but there was no significant difference between MAP and MAP(Ifo) (or MAP plus etoposide). This meta-analysis, however, contained only two phase III randomized studies that compared regimens with HDMtx and a non-HDMtx regimen, the outcome of which was negative.

Thus the use of HDMtx is largely supported by phase II studies and the vast experience that centers have with use of this regimen, rather than from randomized controlled trials comparing HDMtx- with non-HDMtx–containing regimens; hence its benefit is not an unquestionable fact. The recent use of HDMtx-containing regimens in randomized trials gives more certainty and reliability regarding the efficacy of methotrexate and may be the reason why non-HDMtx regimens are not often used in clinical trials.^11,12^ To our knowledge, there has been no randomized trial to compare non-HDMtx triple-drug therapy with HDMtx-containing triple-drug therapy such as ifosfamide, adriamycin, and cisplatin.

## WHY HDMtx CHEMOTHERAPY IS NOT CUT OUT FOR INDIAN SCENARIOS

The use of HDMtx requires admission, rigorous hydration, and leucovorin rescue with the associated toxicities. In most of the tertiary care hospitals in India, the logistic issues do not allow administration of HDMtx because of difficulty in inpatient admissions (and associated cost) and lack of facilities to measure methotrexate levels. HDMtx is associated with a 4% risk of renal failure, and an associated 2% mortality risk in developed countries.^13^ This is compounded by the fact that most of the hospitals in India do not have hemodialysis facilities for timely discovery of the most-feared toxicity of HDMtx—renal failure. Furthermore, carboxypeptidase G2, which is the rescue drug for methotrexate intoxication, is not available in India. This emphasizes the need for chemotherapy according to the available resources, without compromising efficacy and with the least possible long- and short-term toxicity.

## RISE OF NON-HDMtx–CONTAINING THREE-DRUG REGIMENS

In light of the above-mentioned problems, non-HDMtx–containing regimens have been frequently used in resource-constrained settings. In recent years, there has been an upsurge in the use and thus publications of non-HDMtx–containing three-drug regimens, with good outcomes in nonmetastatic OS. The majority of studies with non-HDMtx regimens used a backbone of cisplatin and doxorubicin with ifosfamide or cyclophosphamide (Table 1). These studies have small sample sizes and variable lengths of follow-up. In the largest of these trials, a multi-institutional study by Daw et al,^14^ doxorubicin and ifosfamide were combined with carboplatin instead of cisplatin, with a resultant 5-year event-free survival rate of 66%. This compares favorably with the outcome seen in an HDMtx-containing chemotherapy regimen.^11^ Furthermore, good outcomes with carboplatin need further trials to confirm its activity as part of a multidrug regimen. It would also decrease the need for hydration (as in cisplatin therapy) and lessen the risks of electrolyte imbalance, nephrotoxicity, and hearing loss. Therefore, in given conditions, an optimal level of care can be delivered by giving multidrug chemotherapy that includes carboplatin, adriamycin, and ifosfamide as backbone. However, it needs further confirmation in a larger sample size.

**Table 1:**
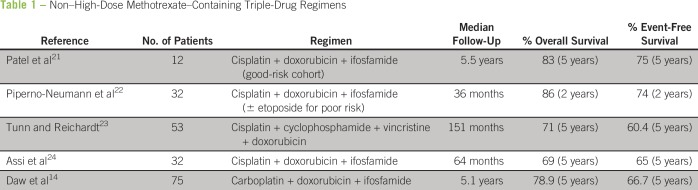
Non–High-Dose Methotrexate–Containing Triple-Drug Regimens

## DOES THE ADDITION OF A FOURTH DRUG ADD BENEFIT TO A THREE-DRUG REGIMEN?

Regarding the addition of ifosfamide to MAP chemotherapy (ie, four-drug chemotherapy), it seems that there is no benefit, as shown by the randomized control trial, Intergroup Study 0133.^11^ This finding was further supported by the meta-analysis by Anninga et al.^10^ Thus, currently there is no beneficial role of adding ifosfamide to MAP chemotherapy.

## DO WE NEED TO CHANGE CHEMOTHERAPY ON THE BASIS OF HISTOLOGIC RESPONSE TO NEOADJUVANT CHEMOTHERAPY?

Percentage necrosis assessment after neoadjuvant chemotherapy is one of the most robust prognostic markers for survival outcome in nonmetastatic OS.^15,16^ Those patients who have > 90% necrosis have a 5-year event-free survival rate of approximately 70% to 80% vis-a-vis 40% to 60% in those with < 90% necrosis.^16,17^ There have been several attempts to change the treatment on the basis of response to chemotherapy. The most successful of these has been the addition of ifosfamide and etoposide in poor responders, which has been shown to improve outcomes in phase II studies.^18^ The EURAMOS trial addressed this question by randomly assigning the high-risk group to continuation of MAP with or without ifosfamide and etoposide. The recently published results of the EURAMOS-1 trial show that the addition of ifosfamide and etoposide in poor responders increased toxicity without any significant benefit.^12^ Furthermore, these patients reported higher rates of second malignant neoplasm and were less likely to complete therapy. This question has not been adequately addressed in patients receiving nonmethotrexate-based regimens. But we do not think that such a collaboration to answer this question is possible in the near future.

## INDIAN DATA: LESSONS TO LEARN

Recently, Nataraj et al^19^ published data for nonmetastatic OS treated with a non-HDMtx regimen in 237 patients from a tertiary care center. Patients received three cycles of neoadjuvant cisplatin and doxorubicin and underwent local surgery. On the basis of percentage necrosis, they received either three cycles of cisplatin and doxorubicin (if necrosis was > 90%) or they received ifosfamide and etoposide alternated with cisplatin and doxorubicin for the next eight cycles if they were poor responders. Thus a few patients received a two-drug regimen (25%), whereas the remaining received a four-drug regimen. After a median follow-up of 30 months, event-free survival and overall survival rates at 5 years were 36% and 50%, respectively. In addition, necrosis did not prove to be a prognostic factor for the final outcome. Such outcomes can be explained by an unusually large tumor size, the inclusion of patients with poor performance status, the use of non-HDMtx–based chemotherapy, or the use of double-drug therapy. The absence of necrosis as a prognostic factor could be explained by the complex and time-consuming method (including decalcification and meticulous pathologic assessment), and thus might lack accuracy in locations that do not have sarcoma pathologists. In a disease such as OS, the quality and timing of surgery should also be evaluated in detail, because surgery is as important as chemotherapy in deciding the outcome. However, all of these factors need to be systematically addressed in a prospective study. Another problem that emerged in the study was that the abandonment rate was approximately 20%, which underscores the fact that in India we have different issues to deal with. In the future, the focus of ongoing research in developing countries should also include the potential requirement of having a strong referral system and improved ancillary services, which is the stepping stone for improved outcomes in OS.

## WHAT NEEDS TO BE DONE? TRIALS AND COLLABORATIONS DIRECTED TO LOCAL NEEDS

There is an urgent need to study non-HDMtx regimens in India or other developing countries with limited resources for feasibility, response rates, toxicities (both long term and short term), and survival outcome in a prospective fashion. In addition, only an adequately powered randomized trial comparing a methotrexate-containing and a nonmethotrexate-containing regimen (preferably using a three-drug regimen for both) will be able to solve the conundrum of the ideal regimen to use in a resource-constrained setting. Second, in places where double-drug therapy is still the regimen of choice, a well-conducted randomized study could address the question of double- versus three-drug therapy. We can take a cue from recent publications that in the developing world, the choice of an appropriate control can be according to local policies and resources and cannot be copied from those used in the developed world.^20^ However, it would require a large sample size and plenty of collaboration. This might be possible only by a EURAMOS-like collaboration between the largest centers in the country. Because this remains the only hope for our patients with OS, we must not forget:

“Unity is strength...when there is teamwork and collaboration, wonderful things can be achieved.”

—Mattie Stepanek

## References

[1] Luetke A Meyers PA Lewis I et al: Osteosarcoma treatment—Where do we stand? A state of the art review. Cancer Treat Rev 40:523-532, 2014 24345772 10.1016/j.ctrv.2013.11.006

[2] Isakoff MS Bielack SS Meltzer P et al: Osteosarcoma: Current treatment and a collaborative pathway to success. J Clin Oncol 33:3029-3035, 2015 26304877 10.1200/JCO.2014.59.4895 PMC4979196

[3] Jaffe N Gorlick R: High-dose methotrexate in osteosarcoma: Let the questions surcease—Time for final acceptance. J Clin Oncol 26:4365-4366, 2008 18802144 10.1200/JCO.2007.14.7793

[4] Bielack SS Smeland S Whelan JS et al: Methotrexate, doxorubicin, and cisplatin (MAP) plus maintenance pegylated interferon alfa-2b versus MAP alone in patients with resectable high-grade osteosarcoma and good histologic response to preoperative MAP: First results of the EURAMOS-1 good response randomized controlled trial. J Clin Oncol 33:2279-2287, 2015 26033801 10.1200/JCO.2014.60.0734 PMC4486345

[5] Edmonson JH Green SJ Ivins JC et al: A controlled pilot study of high-dose methotrexate as postsurgical adjuvant treatment for primary osteosarcoma. J Clin Oncol 2:152-156, 1984 6366150 10.1200/JCO.1984.2.3.152

[6] Link MP Goorin AM Miser AW et al: The effect of adjuvant chemotherapy on relapse-free survival in patients with osteosarcoma of the extremity. N Engl J Med 314:1600-1606, 1986 3520317 10.1056/NEJM198606193142502

[7] Bramwell VH Burgers M Sneath R et al: A comparison of two short intensive adjuvant chemotherapy regimens in operable osteosarcoma of limbs in children and young adults: The first study of the European Osteosarcoma Intergroup. J Clin Oncol 10:1579-1591, 1992 1403038 10.1200/JCO.1992.10.10.1579

[8] Souhami RL Craft AW Van der Eijken JW et al: Randomised trial of two regimens of chemotherapy in operable osteosarcoma: A study of the European Osteosarcoma Intergroup. Lancet 350:911-917, 1997 9314869 10.1016/S0140-6736(97)02307-6

[9] van Dalen EC van As JW de Camargo B: Methotrexate for high-grade osteosarcoma in children and young adults. Cochrane Database Syst Rev (5):CD006325, 2011 19160278 10.1002/14651858.CD006325.pub2

[10] Anninga JK Gelderblom H Fiocco M et al: Chemotherapeutic adjuvant treatment for osteosarcoma: Where do we stand? Eur J Cancer 47:2431-2445, 2011 21703851 10.1016/j.ejca.2011.05.030

[11] Meyers PA Schwartz CL Krailo MD et al: Osteosarcoma: The addition of muramyl tripeptide to chemotherapy improves overall survival—A report from the Children’s Oncology Group. J Clin Oncol 26:633-638, 2008 18235123 10.1200/JCO.2008.14.0095

[12] Marina NM Smeland S Bielack SS et al: Comparison of MAPIE versus MAP in patients with a poor response to preoperative chemotherapy for newly diagnosed high-grade osteosarcoma (EURAMOS-1): An open-label, international, randomised controlled trial. Lancet Oncol 17:1396-1408, 2016 27569442 10.1016/S1470-2045(16)30214-5 PMC5052459

[13] Widemann BC Adamson PC: Understanding and managing methotrexate nephrotoxicity. Oncologist 11:694-703, 2006 16794248 10.1634/theoncologist.11-6-694

[14] Daw NC Neel MD Rao BN et al: Frontline treatment of localized osteosarcoma without methotrexate: Results of the St. Jude Children’s Research Hospital OS99 trial. Cancer 117:2770-2778, 2011 21656756 10.1002/cncr.25715 PMC3535449

[15] Bakhshi S Radhakrishnan V: Prognostic markers in osteosarcoma. Expert Rev Anticancer Ther 10:271-287, 2010 20132002 10.1586/era.09.186

[16] Bielack SS Kempf-Bielack B Delling G et al: Prognostic factors in high-grade osteosarcoma of the extremities or trunk: An analysis of 1,702 patients treated on neoadjuvant Cooperative Osteosarcoma Study Group protocols. J Clin Oncol 20:776-790, 2002 11821461 10.1200/JCO.2002.20.3.776

[17] Bacci G Bertoni F Longhi A et al: Neoadjuvant chemotherapy for high-grade central osteosarcoma of the extremity. Histologic response to preoperative chemotherapy correlates with histologic subtype of the tumor. Cancer 97:3068-3075, 2003 12784343 10.1002/cncr.11456

[18] Bacci G Longhi A Versari M et al: Prognostic factors for osteosarcoma of the extremity treated with neoadjuvant chemotherapy: 15-year experience in 789 patients treated at a single institution. Cancer 106:1154-1161, 2006 16421923 10.1002/cncr.21724

[19] Nataraj V Batra A Rastogi S et al: Developing a prognostic model for patients with localized osteosarcoma treated with uniform chemotherapy protocol without high dose methotrexate: A single-center experience of 237 patients. J Surg Oncol 112:662-668, 2015 26381138 10.1002/jso.24045

[20] Gupta S Gulia S Shetty N: Clinical trial ethics: One standard does not fit all. J Clin Oncol 33:1413-1414, 2015 25713433 10.1200/JCO.2014.59.5371

[21] Patel SJ Lynch JW Jr Johnson T et al: Dose-intense ifosfamide/doxorubicin/cisplatin based chemotherapy for osteosarcoma in adults. Am J Clin Oncol 25:489-495, 2002 12393991 10.1097/00000421-200210000-00014

[22] Piperno-Neumann S, Bui B, Blay J, et al: A multicentric prospective study of intensive induction chemotherapy (API-AI) in localized osteosarcoma patients: Results of a phase II trial coordinated by the French Sarcoma Group (FSG) and the FNCLCC BECT. J Clin Oncol 24:9521, 2006

[23] Tunn PU Reichardt P: Chemotherapy for osteosarcoma without high-dose methotrexate: A 12-year follow-up on 53 patients. Onkologie 30:228-232, 2007 17460416 10.1159/000100776

[24] Assi H Missenard G Terrier P et al: Intensive induction chemotherapy without methotrexate in adult patients with localized osteosarcoma: Results of the Institut Gustave-Roussy phase II trial. Curr Oncol 17:23-31, 2010 21151406 10.3747/co.v17i6.578 PMC2993436

